# Therapy for aggressive pituitary tumors and carcinomas

**DOI:** 10.1007/s11154-026-10039-4

**Published:** 2026-04-17

**Authors:** Mirela-Diana Ilie, Elisa Lamback, Mônica Gadelha, Gérald Raverot

**Affiliations:** 1 “Carol Davila” University of Medicine and Pharmacy, Bucharest, Romania; 2 Universidade Federal do Rio de Janeiro, Rio de Janeiro, Brazil; 3 Instituto Estadual do Cérebro Paulo Niemeyer, Rio de Janeiro, Brazil; 4 Inserm U1052, CNRS UMR5286, Claude Bernard Lyon 1 University, Cancer Research Center of Lyon, Lyon, France; 5 Endocrinology Department, Reference Center for Rare Pituitary Diseases HYPO, “Groupement Hospitalier Est” Hospices Civils de Lyon, Bron, France

**Keywords:** Aggressive pituitary tumor, Pituitary carcinoma, Temozolomide, Immune-checkpoint inhibitors, Bevacizumab, Peptide receptor radionuclide therapy

## Abstract

The management of aggressive pituitary tumors and carcinomas remains challenging. This review article discusses available treatments in relation to the recently published Revised European Society of Endocrinology Clinical Practice Guideline for the management of aggressive pituitary tumors and pituitary carcinomas. Specifically, we discuss both established treatment options, with a focus on temozolomide, and emerging treatment options. Regarding the emerging treatment options, we focus on molecular-guided druggable targets, immune-checkpoint inhibitors, bevacizumab and peptide receptor radionuclide therapy. For each treatment option, we discuss tumor response, predictive factors of response to treatment, adverse events, as well as perspectives.

**Graphical Abstract:**

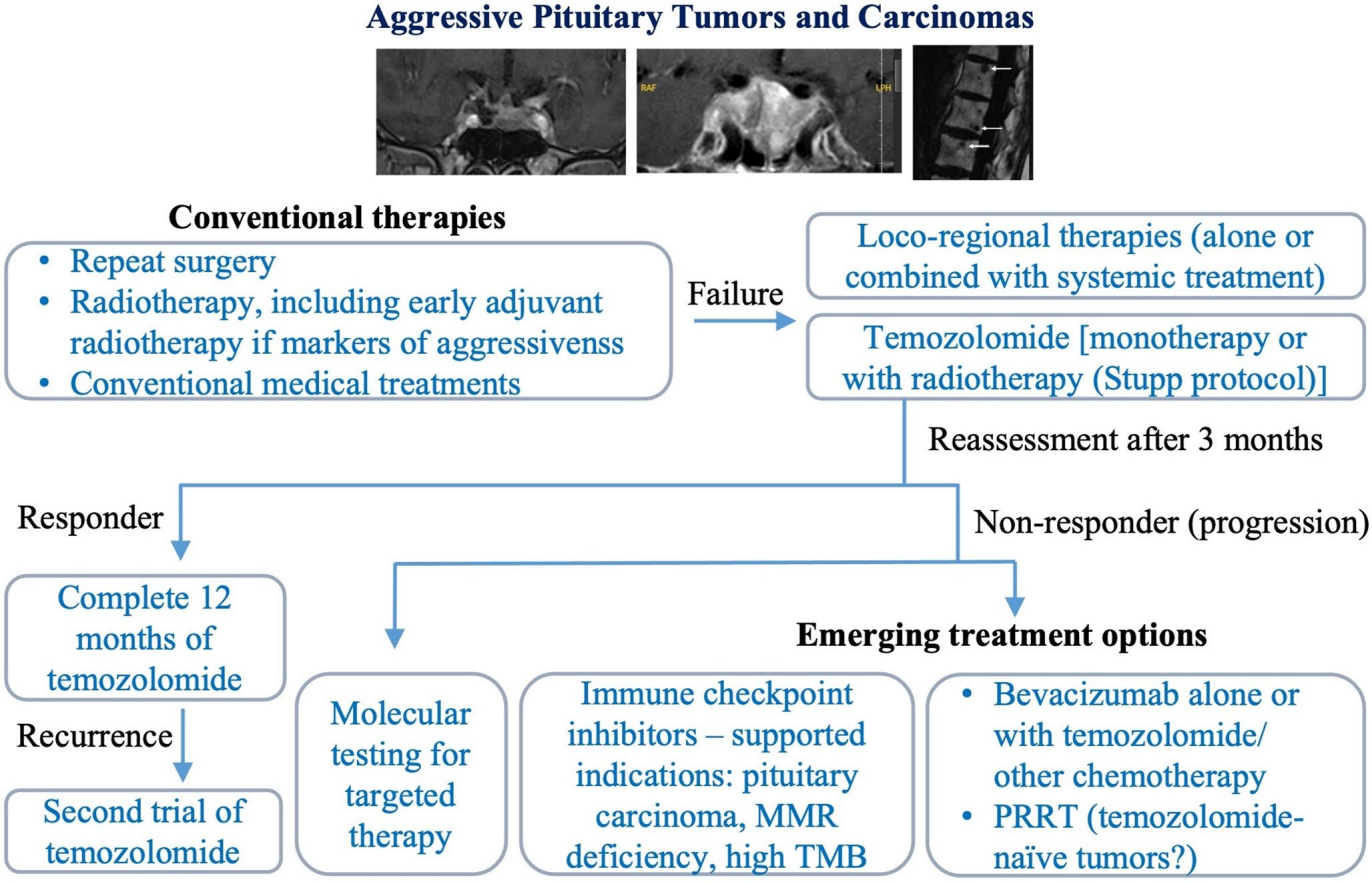

## Introduction

According to the revised European Society of Endocrinology clinical practice guideline for the management of aggressive pituitary tumors and pituitary carcinomas [1], the diagnosis of an aggressive pituitary tumor, also named aggressive pituitary neuroendocrine tumor (PitNET), should be considered in patients with a radiologically-invasive tumor, and either unusually rapid tumor growth rate, or clinically relevant tumor growth despite optimal standard therapies; standard therapies include surgery, radiotherapy, and conventional medical treatments, i.e., somatostatin receptor ligands and dopamine type 2 receptor agonists. Pituitary carcinomas, also named metastatic PitNETs, are very rare and can be diagnosed only in the presence of craniospinal or systemic metastases [1, 2]. From a practical perspective, defining pituitary carcinomas by the presence of metastasis makes the extension work-up critical not only for guiding the treatment strategy, but also for establishing the diagnosis.

Importantly, metastases may be detected at any time, ranging from several months to many years following the initial diagnosis [3, 4]. However, predicting future aggressive behavior remains challenging. It was speculated that 2B* tumors (defined as invasive and highly proliferative tumors, with a Ki67 index ≥ 10%) might be suspect of malignancy [5, 6]. Along the same lines, it was also proposed that an initial Ki67 index ≥ 10% might be an early marker of aggressiveness [3]. Other markers associated with aggressive pituitary tumors and carcinomas include somatic pathogenic variants with *ATRX* and *TP53* pathogenic variants being encountered especially in corticotroph tumors, and *SF3B1* and *TP53* pathogenic variants in lactotroph tumors [1, 7, 8]; aneuploidy due to widespread chromosomal loss of heterozygosity was also reported in corticotroph carcinomas and aggressive pituitary tumors [9].

Treating patients with aggressive pituitary tumors and carcinomas remains equally challenging. We have recently reviewed therapies targeting the pituitary tumor microenvironment [10] and treatment options beyond temozolomide [11]. This review article will discuss available treatments in relation to the revised guidelines [1], including both established (notably temozolomide) and emerging treatment options.

## Established treatment options

### Conventional therapies

Although we will not extensively discuss this part, it is important to bear in mind that the revised guidelines recommend as therapy for aggressive pituitary tumors and carcinomas (a) repeat surgery performed by an expert neurosurgeon, (b) radiotherapy, as radiotherapy, adjuvant radiotherapy, and/or a second course of radiotherapy, as well as (c) maximally tolerated doses of conventional pituitary-directed medical treatments: somatostatin receptor ligands for somatotroph, corticotroph and thyrotroph tumors, and cabergoline for lactotroph and somatotroph tumors [1].

Of note, even before proved aggressive behavior, the guidelines suggest considering early adjuvant radiotherapy for patients with residual tumor in the presence of pathological or molecular markers associated with aggressiveness. Specifically, the guidelines include here grade 2B* tumors, lactotroph tumors resistant to dopamine type 2 receptor agonists and bearing *SF3B1* or *TP53* pathogenic variants, and macrocorticotroph tumors bearing *ATRX* or *TP53* pathogenic variants [1].

### Temozolomide

Both the initial and the revised guidelines recommend temozolomide, an oral alkylating therapy, as the first-line treatment for aggressive pituitary tumors and carcinomas after standard therapy failure and following documented tumor progression [1, 12].

The revised guidelines recommend temozolomide monotherapy for 12 months in responders. A longer duration of treatment could be considered to obtain a maximal response in the absence of side effects. In non-responders with tumor progression demonstrated after three cycles, temozolomide treatment should be ceased [1]. The Stupp protocol, i.e., temozolomide combined with radiotherapy, followed by temozolomide alone appears to have a higher response rate in aggressive pituitary tumors and carcinomas, although data remains limited [1, 13]. It could be considered in rare cases of rapid tumor progression of a large residual tumor, inaccessible to additional surgery, particularly in the presence of pathological or molecular markers suggestive of a poor prognosis, or when a rapid tumor response is required [1].

Temozolomide remains first-line therapy because it has a reasonable effectiveness with around 40% of patients showing complete or partial radiological response and one third of patients showing stable disease [4, 14]. However, a third of patients progress on first-line temozolomide and, even if the initial response is encouraging, after stopping temozolomide, an important proportions of tumors may recur or progress again [4, 15]. Moreover, a second trial of temozolomide is less effective [4, 15]. Nevertheless, the revised guidelines suggest a second trial of temozolomide in patients with recurrence following prior complete or partial response to the first course of temozolomide [1]. We illustrate hereafter the good effectiveness of a first and second course of temozolomide.

So far, the only potential predictor of response to temozolomide in aggressive pituitary tumors and carcinomas appears to be the methylguanine methyltransferase (MGMT) status, however inconsistent results have been reported [1, 15–18]. It is no longer recommended to perform MGMT immunohistochemistry before a trial of temozolomide.

Regarding the histological type and secretory status, the first European Society of Endocrinology survey concluded that functioning tumors responded better to temozolomide, without further difference among histological types of functioning tumors [15]. Similarly, a meta-analysis identified better response to temozolomide in functioning compared with non-functioning tumors [14].

Regarding adverse events, temozolomide is associated with both acute and late toxicities [19]. The revised guidelines note that treatment duration with temozolomide exceeding 24 months must be weighed against the potential risk for cumulative severe toxicity [1], including secondary malignancies. In addition, both in gliomas and in aggressive pituitary tumors and carcinomas, use of temozolomide is associated, at least in some patients, with temozolomide-induced hypermutation [20, 21]. While temozolomide-induced hypermutation may render immune-checkpoint inhibitors (ICIs) more effective [21, 22], it raises the question of whether alternative treatments should be considered for aggressive pituitary tumors before using temozolomide.

Importantly, in low-grade gliomas, temozolomide-induced hypermutation was demonstrated to be associated on the one hand with high-grade histology at recurrence, and on the other hand, with both increased risk of distant recurrence and reduced survival after high-grade transformation [20]. Whether these associations apply also to aggressive pituitary tumors and carcinomas has not been investigated yet.

Thus, perspectives regarding temozolomide include improving its effectiveness (for example by combining it with radiotherapy or other drugs, and by identifying predictors of response to temozolomide), as well as potentially reconsidering its role as the primary therapy for aggressive pituitary tumors and carcinomas.

### Loco-regional therapies

If oligo-metastatic disease in present, the revised guidelines suggest loco-regional therapies, such as surgery, radiotherapy, radiofrequency or microwave ablation, chemoembolization or bland embolization, to be used either alone or combined with systemic treatment [1].

## Emerging treatment options

After temozolomide failure, the initial guidelines suggested a trial with other systemic cytotoxic therapy. However, given the variety of chemotherapeutic agents that had been reported and the lack of evidence, they were unable to suggest a particular regimen at that time [12]. Since, more data has accumulated which led to significant changes in the revised guidelines [1], as discussed below.

### Molecular-guided druggable targets

First, to guide potential treatment choices, the revised guidelines suggest molecular testing in patients with tumor progression on temozolomide [1]. This molecular testing may provide information on specific gene alterations that can be targeted therapeutically, but also additional information, such as the tumor mutational burden and the mismatch repair deficiency status, for example [1].

Regarding the utility of molecular profiling in aggressive pituitary tumors and carcinomas, in a recent study, Paes et al. sequenced a cohort of 20 patients with prolactinomas to identify somatic genetic alterations. By doing so, they identified a somatic activating mutation in the estrogen receptor alpha gene in a patient with an aggressive lactotroph tumor resistant to temozolomide. This led to treatment with elacestrant, a second-line estrogen-receptor degrader. Elacestran was initiated after the third course of radiotherapy was completed and managed to significantly reduce prolactin levels and to control tumor growth for the available follow-up [23].

Another important point is that the respective somatic mutation, while present on the tissue sample from the fourth surgery, it was absent on the tissue samples from the first and second surgeries [23], underlying the need of a recent tissue sample for molecular profiling. In addition, Lin et al. demonstrated that different lesions from a same patients may harbor different molecular profiles, that may influence the response to treatment, more specifically to ICIs in the respective study [21].

In line with this, in the revised guidelines, it is noted that performing genomic analysis on recent tumor tissue is important, and hence to consider minimal invasive surgical biopsy as it poses minimal risk [1]. Having tissue available from different tumor sites in the case of pituitary carcinomas appears equally important.

### Immunotherapy

Second, among emerging treatment options, ICIs have gained the most attention in recent years. The revised guidelines suggest considering a trial with ICIs in patients with pituitary carcinoma and rapid tumor progression after treatment with temozolomide. They also note that tumor agnostic data supports the use of ICIs in tumors that are either mismatch repair deficient or exhibit high tumor mutational burden, supporting the use in pituitary tumors with these molecular features [1].

By blocking immune checkpoints, “the brakes” of the immune system, ICIs reactivate and enhance anti-tumoral immune responses [24]. More specifically, ICIs used so far for the treatment of pituitary tumors block cytotoxic T-lymphocyte-associated protein (CTLA4) in the case of ipilimumab, and programmed cell death protein 1 (PD-1) in the case of nivolumab and pembrolizumab [10]. These molecules were used either as combined therapy (nivolumab + ipilimumab) or as monotherapy – usually pembrolizumab, but nivolumab monotherapy was also used [10].

The tumor response of 38 pituitary cases has so far been reported. These cases come from a cohort of 15 cases [25], a phase 2 clinical trial of nine cases [21], another phase 2 clinical trial of four cases [26], while the others are cases from the second European Society of Endocrinology survey [4] or from isolated case reports [11, 22, 27–32]. All 38 patients received multimodal treatment before ICI start, including temozolomide in at least 36 cases [4, 11, 21, 22, 25–32].

**Fig. 1:**
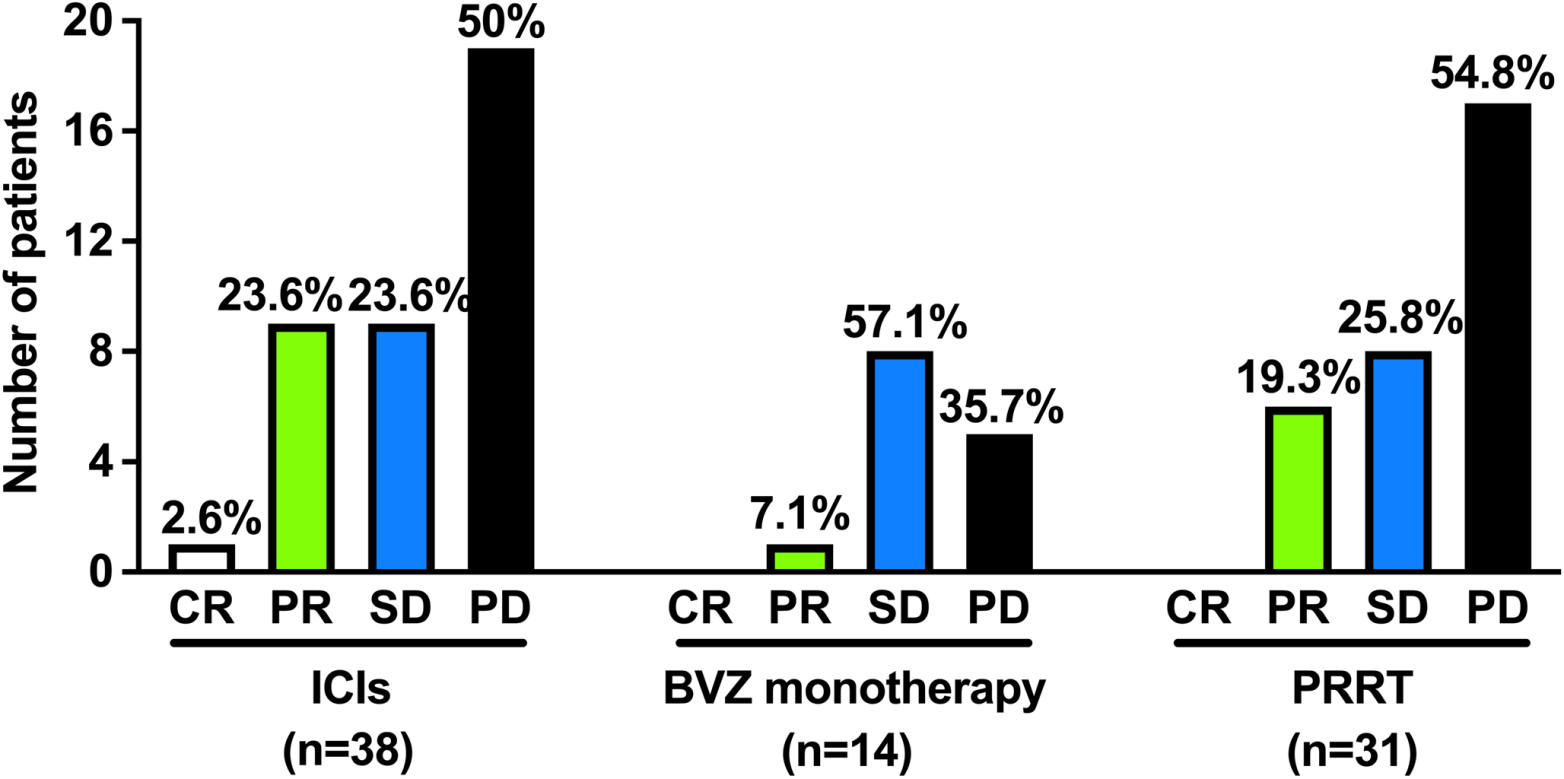
Tumor response in aggressive pituitary tumors and carcinomas. Abbreviations: immune-checkpoint inhibitors (ICIs), bevacizumab (BVZ), peptide receptor radionuclide therapy (PRRT), complete response (CR), partial response (PR), stable disease (SD), progressive disease (PD). Made with Graphpad Prism version 10.6.1

**Fig. 2:**
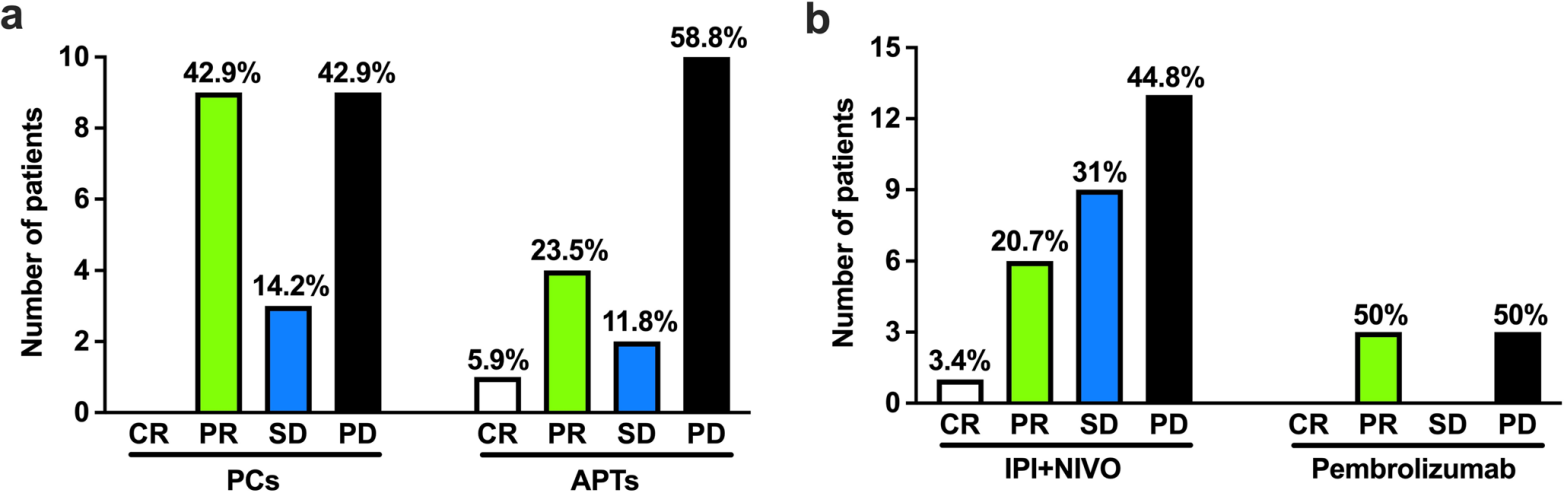
Tumor response to immune-checkpoint inhibitors in aggressive pituitary tumors and carcinomas considered separately for pituitary carcinomas versus aggressive pituitary tumors (**a**), as well as separately for dual therapy versus monotherapy with pembrolizumab (**b**). Abbreviations: pituitary carcinomas (PCs), aggressive pituitary tumors (APTs), ipilimumab (IPI), nivolumab (NIVO), complete response (CR), partial response (PR), stable disease (SD), progressive disease (PD). Made with Graphpad Prism version 10.6.1

Under ICIs, complete response was noted in one case (2.6%) [32], partial response in nine cases (23.6%), stable disease in nine cases (23.6%), while 19 (half) of the tumors progressed [4, 11, 21, 22, 25–32] (Fig. 1). Figure 1 shows the tumor response for all cases, while Fig. 2a the response separately for pituitary carcinomas versus aggressive pituitary tumors, and Fig. 2b the response separately for dual therapy versus monotherapy with pembrolizumab. However, as with all treatments, one should bear in mind that different studies or case reports have evaluated differently the tumor response, and that the duration of the response is also variable [4, 11, 21, 22, 25–32].

Regarding predictors of response to treatment, in our cohort study we identified that tumor type (i.e., pituitary carcinoma versus aggressive pituitary tumor) was a major predictor of response to ICIs, with pituitary carcinomas responding better. With the publication of more data, although this difference persists, it appears to become less pronounced (Fig. 2a).

As noted in the revised guidelines, mismatch repair deficiency and high tumor mutational burden are known predictors in other cancers [1]. Tumor mutational burden appears promising as a predictive factor of response in aggressive pituitary tumors and carcinomas, as well; however, it is noteworthy that a high tumor mutational burden has been associated with a favorable radiological response in most, but not all treated cases [4, 10, 11, 13, 21]. Similarly, although mismatch repair deficiency appears promising, not all mismatch repair-deficient cases responded to ICIs [10, 21].

Regarding the histological type, the cases treated consist of 24 corticotroph tumors, 12 lactotroph tumors, one Pit1 silent tumor and a somatotroph tumor [4, 11, 21, 22, 25–32]. One corticotroph tumor showed complete response, six (25%) partial response, three (12.5%) stable disease, while 14 (58.3%) progressed. On the other hand, two (16.7%) lactotroph tumors showed partial response, five (41.7%) stable disease, while the other five (41.7%) progressed. Thus, it is difficult to say whether tumor histological type might play a role.

Finally, only four patients with corticotroph tumors had hypercortisolism during ICIs administration [4, 10, 11, 21, 22, 25–32]. The numbers are too low to draw any definitive conclusion, nonetheless, given the immunomodulatory effect of glucocorticoids, it appears prudent to aim for eucortisolism.

Regarding immune-related adverse events, as reported also for other cancers, more frequent and more severe adverse events occurred when using combined immunotherapy versus monotherapy [4, 11, 21, 22, 25–32]. Notably, under combined ICIs, they ranged from mild to severe or even life-threatening [4, 11, 21, 22, 25–32]. Since, in addition, it does not appear so far to be a clear advantage when comparing the effectiveness of combined ipilimumab and nivolumab versus monotherapy (especially pembrolizumab monotherapy) [1, 10, 11], the revised guidelines consider these to be arguments for the initial treatment with PD-1 blockers as monotherapy [1]. However, as shown in Fig. 2b, which illustrates the updated comparison between combined ICIs versus pembrolizumab monotherapy [11, 21], only six cases treated with pembrolizumab alone are available in literature.

Regarding treatment duration, as with all treatments, this should be a multidisciplinary decision based on effectiveness and tolerability. In our practice, we administer four cycles of combined ipilimumab and nivolumab, followed by maintenance nivolumab for as long as it is well tolerated and remains effective. Similarly, pembrolizumab monotherapy, might be continued for as long as it is well tolerated and remains effective. This generally means until progressive disease is noted. However, in cases of a dissociate response between lesions (in case of pituitary carcinoma), additional treatments may be added without stopping immunotherapy.

There are two main perspectives regarding immunotherapy: improving the effectiveness of ICIs, and using other types of immunotherapies – for example, targeting tumor-associated macrophages [33] or mast cells [34]. Regarding the improvement of ICIs’ effectiveness, potential leads include combination therapies – for example, with radiotherapy, peptide receptor radionuclide therapy (PRRT) or anti-angiogenic drugs [27, 35, 36]. Identification and validation of predictors of response, and thus, the personalized use of immune-checkpoint inhibitors, will also results in improved observed effectiveness.

### Other treatment options

Finally, given the limited data supporting the use of cytotoxic chemotherapy other than temozolomide and of targeted agents, if access to ICIs is difficult and in the absence of a molecular-guided druggable target, the revised guidelines recommend participation in clinical studies [1]. Nevertheless, on their algorithm figure, they include PRRT for tumors with high standardized uptake value (SUV) on 68Ga-DOTA-TOC or 68Ga-DOTA-TATE PET; otherwise, bevacizumab and/or conventional chemotherapy [1]. Thus, we will discuss below bevacizumab and PRRT.

#### Bevacizumab

Bevacizumab, a monoclonal antibody targeting VEGF-A, has been the most commonly used antiangiogenic drug for the treatment of aggressive pituitary tumors and carcinomas [10, 11]. The tumor response is available for 26 aggressive pituitary tumors and carcinomas [4, 11, 15, 26, 29, 37–45], of which 14 patients treated with bevacizumab monotherapy (Fig. 1). No clinical trial or large cohort is available for bevacizumab.

Regarding monotherapy, all 14 patients had previously received temozolomide; most (57.1%) of these 14 patients [4, 15, 29, 40–45] showed stable disease. There was only one case classified as partial response but even in this case, there was partial response of the pituitary lesion, while the metastases showed stable disease [4].

For the four cases treated with bevacizumab combined with first-line temozolomide [15, 37–39], the response is quite impressive (one complete response, two partial responses and one stable disease), but temozolomide itself has a quite good effectiveness. Of note, in the case of the complete response, bevacizumab was combined with a Stupp protocol [37].

When looking at the response to bevacizumab combined with a second course of temozolomide [4, 15], we encounter one partial response, one stable disease and two progressive diseases. Considering that a second course of temozolomide is more rarely effective, we may get the impression that bevacizumab add-on could provide some benefit. However, this remains to be confirmed on a higher number of patients. Finally, too few cases (four) treated with bevacizumab combined with other chemotherapies [4, 11, 26] are reported to draw any conclusion.

VEGF-A/VEGF receptor immunoreactivity and microvessel density have been associated with a superior response to bevacizumab in a variety of cancers [46–49]. Unfortunately, data regarding the expression of VEGF and/or VEGF receptors in the aggressive pituitary tumors and carcinomas treated with bevacizumab have been rarely reported and have been inconclusive so far. No data is available on the microvessel density in the treated cases [10].

The presence or absence of adverse events was rarely reported, and included fatigue, epistaxis, petechiae, hypertension, nephritis and cellulitis [11].

Regarding treatment duration, as with immunotherapy, in our practice, we administer bevacizumab for as long as it is well tolerated and remains effective.

Similar to immunotherapy, there are two main perspectives: enhancing bevacizumab’s effectiveness and exploring alternative antiangiogenic therapies. To boost bevacizumab’s performance, strategies such as combining it with chemotherapy or immunotherapy might be considered [36]. Additionally, identifying response predictors for personalized bevacizumab use could further increase its observed effectiveness.

#### PRRT

The principle of PRRT used in aggressive pituitary tumors and carcinomas is the following: a therapeutic radiopharmaceutical (^177^Lutetium, ^90^Yttrium or ^68^Gallium, of which ^177^Lutetium has been the most used so far) is linked to a somatostatin receptor ligand (^177^Lutetium was conjugated either with TATE or TOC) by a binding chelator (for example, DOTA) [11, 50].

**Fig. 3:**
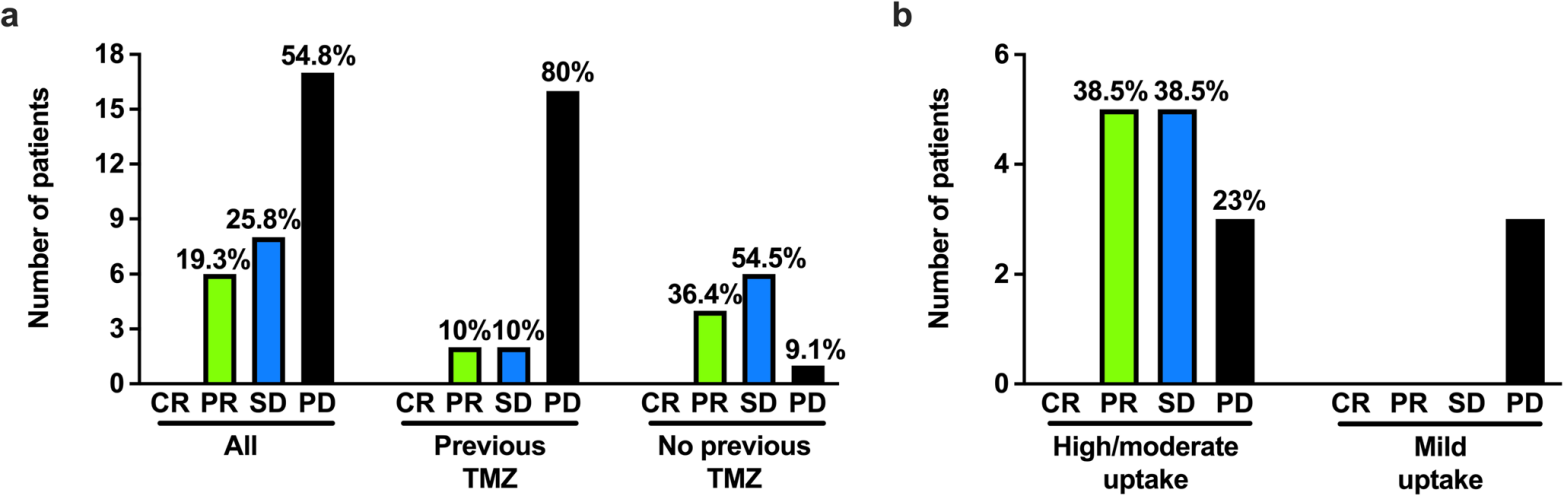
Tumor response to peptide receptor radionuclide therapy in aggressive pituitary tumors and carcinomas considered both together and separately in relation to previous temozolomide treatment (**a**) and in relation with the uptake on functional imaging (**b**). Abbreviations: complete response (CR), partial response (PR), stable disease (SD), progressive disease (PD), temozolomide (TMZ). Made with Graphpad Prism version 10.6.1

The tumor response, available for 31 aggressive pituitary tumors and carcinomas [4, 11, 15, 16, 26, 27, 43, 51–61], is summarized in Fig. 3a, together for all patients, and separately for the 20 patients previously treated with temozolomide versus the 11 patients without previous temozolomide treatment. Of note, no clinical trial or large cohort is available for PRRT.

When considering all cases together, partial response was obtained in six cases (19.3%), eight (25.8%) other patients showed stable disease, while most (54.8%) tumors progressed [4, 11, 15, 16, 26, 27, 43, 51–61]. However, as seen in Fig. 3a, temozolomide-naïve tumors showed a much better response, with most tumors (six; 54.5%) showing stable disease, four (36.4%) partial response and only one (9.1%) tumor showing progressive disease. This better response might be due to different factors from publication bias to a potentially less aggressive phenotype of some of the tumors treated with PRRT instead of temozolomide. Nonetheless, whether PRRT should be tried earlier, and maybe even before temozolomide, at least in a subset of aggressive pituitary tumors and carcinomas, is a recurring question [6, 57, 60]. This question is worthy of further studies, especially given that PRRT is currently preferred to chemotherapy at least for a subset of neuroendocrine tumors [62].

Regarding predictors of response, since PRRT works by targeting somatostatin receptors on tumor cell membranes [50], one can expect that tumors must express these receptors for the treatment to be effective. Indeed, it seems to be the case, as seen in Fig. 3b, where we summarize the available data on somatostatin receptor expression (evaluated by functional imaging) in 16 cases treated with PRRT [4, 11, 15, 16, 26, 27, 43, 51–61]. As depicted in Fig. 3b, the tumors that had high or moderate uptake on functional imaging showed a much better response to PRRT, while the 3 patients that received PRRT despite low uptake, showed progressive disease.

Regarding adverse events, PRRT has been generally well tolerated [11]. The most frequent adverse event in the pituitary cases has been transient cytopenia [11, 60, 61]; other notable adverse events included pituitary apoplexy, increase in facial pain and an asymptomatic hemorrhage adjacent to tumor site [11].

The main perspectives include identifying the optimal timing for PRRT, enhancing its observed effectiveness by using a personalized approach, as *well* as potentially increasing the expression of somatostatin receptors on the cell membrane [63] or identifying alternate ligands [64].

## Conclusion

Temozolomide remains the first-line chemotherapeutic agent for aggressive pituitary tumors and carcinomas; however, enhancing its therapeutic effectiveness is of ongoing importance. After temozolomide failure, consideration should be given to molecularly targeted therapies and ICIs, with bevacizumab and PRRT also representing potential alternatives. Additionally, in the presence of metastases, loco-regional treatments should be evaluated regardless of concurrent systemic therapy.

## Data Availability

No datasets were generated or analyzed during the current study.

## Abbreviations

CTLA4: cytotoxic T-lymphocyte-associated protein
ICIs: immune-checkpoint inhibitors
MGMT: methylguanine methyltransferase
PRRT: peptide receptor radionuclide therapy
PitNET: pituitary neuroendocrine tumor
PD-1: programmed cell death protein 1
SUV: standardized uptake value
